# Visible-light-responsive Au-coated NiTi–O nanotubes for enhancing osteogenic differentiation of human mesenchymal stem cells

**DOI:** 10.1039/d6ra04604e

**Published:** 2026-09-24

**Authors:** Kyoung-Suk Moon, Ji-Myung Bae, Gyu-Yeon Shim, Seoung Hoon Lee, Seunghan Oh, Eun-Joo Choi

**Affiliations:** a Department of Dental Biomaterials, College of Dentistry, Wonkwang University Iksan Jeonbuk Republic of Korea shoh@wku.ac.kr +82-63-850-6982; b The Institute of Biomaterials and Implants, Wonkwang University Iksan Jeonbuk Republic of Korea; c Department of Oral Microbiology and Immunology, College of Dentistry, Wonkwang University Iksan Jeonbuk Republic of Korea; d Integrated Omics Institute, Wonkwang University Iksan Jeonbuk Republic of Korea; e Department of Oral and Maxillofacial Surgery, College of Dentistry, Wonkwang University Iksan Jeonbuk Republic of Korea; f Wonkwang Dental Research Institute, College of Dentistry, Wonkwang University Iksan Jeonbuk Republic of Korea

## Abstract

The biological performance of widely used nickel–titanium (NiTi) implants is strongly influenced by nanoscale surface characteristics. This study evaluated Au-coated NiTi–O nanotubes (ANT) as a visible-light-responsive osteogenic interface for human mesenchymal stem cells under 600 nm irradiation. NiTi–O nanotubes were fabricated by anodic oxidation followed by Au sputter coating, which preserved the open nanotubular morphology. Live/dead and MTT assays demonstrated that nanotube fabrication, Au coating, and 600 nm irradiation showed excellent biocompatibility. Under osteogenic conditions, 600 nm irradiation increased ALP activity, particularly on nanotubular surfaces. Among all experimental groups, the 600 nm-irradiated Au-coated nanotube surface showed the highest OPN, OCN, and BSP-2 expression, with the greatest matrix mineralization at 3 weeks. HSP27 and HSP70 were also elevated, consistent with mild photothermal adaptation associated with enhanced visible-to-near-infrared absorption in ANT near 600 and 780 nm. YAP nuclear localization and F-actin organization were primarily associated with nanotopography, whereas irradiation additionally stimulated HSP-related responses. Two-way ANOVA showed an irradiation effect on ALP activity at 1 week, while both surface and irradiation effects were evident at later endpoints. Significant interactions were observed only for ALP (2 weeks), OCN, HSP27, and HSP70. Mineralization showed additive, non-interacting surface (*P* < 0.0001) and irradiation effects (*P* = 0.0010). Collectively, these findings suggest that ANT promotes osteogenic differentiation through nanotopographical and photothermal mechanisms that interact at the level of ALP, OCN, and HSP responses but contribute additively to terminal mineralization.

## Abstract 2

Visible-light-responsive Au-coated NiTi–O nanotubes enhance osteogenic differentiation of hMSCs *in vitro*.

## Graphical abstract

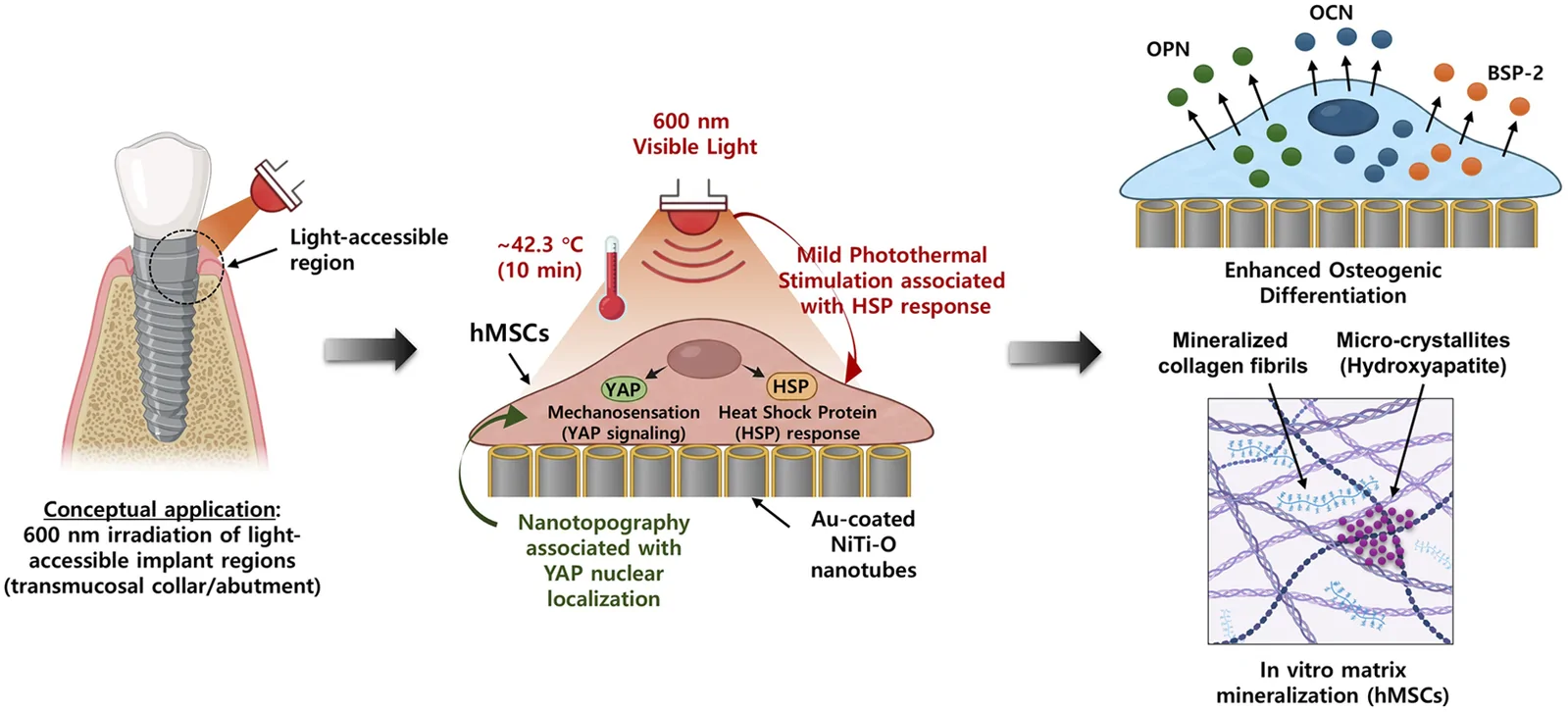

## 1. Introduction

Nickel–titanium (NiTi) alloys are widely used metallic biomaterials in dentistry and orthopedics because of their excellent superelasticity, shape-memory behavior, corrosion resistance, and biocompatibility.^1–3^ However, the clinical performance of metallic implants depends not only on their bulk properties but also on the chemical composition, oxide layer characteristics, surface roughness, and nanostructure of the implant surface that directly interfaces with surrounding cells.^4,5^ Since mesenchymal stem cell attachment, proliferation, osteogenic differentiation, and matrix mineralization are critical during the early stages of osseointegration, surface modification strategies that enhance the biological activity of metallic surfaces are essential.^6,7^

NiTi–O nanotubes fabricated by anodic oxidation provide a high specific surface area and an ordered nanostructure, capable of regulating cell adhesion, cytoskeletal reorganization, and osteogenic signaling pathways.^8,9^ Incorporation of a gold (Au) coating further improves chemical stability and introduces photothermal responsiveness, enabling the conversion of light energy into localized thermal stimulation.^10,11^ Surface plasmon resonance (SPR) refers to the collective oscillation of conduction-band electrons at a metal–dielectric interface when resonantly excited by incident light, producing strong local electromagnetic field enhancement and wavelength-selective light absorption. In noble-metal nanostructures, this resonance enables efficient photothermal conversion and photocatalytic activity in the visible–NIR region.^12–16^ Therefore, owing to their SPR properties, Au-based nanostructures exhibit efficient light absorption and photothermal conversion in the visible and near-infrared regions.^17,18^ Consequently, these nanostructures have attracted considerable interest not only in cancer therapy but also in antimicrobial surface engineering, tissue regeneration, and functional implant design.^19–21^ The size, morphology, and composition of Au nanostructures are therefore considered critical factors governing the light-absorption spectrum and photothermal conversion efficiency.

Previous studies demonstrated that Au-coated NiTi–O nanotube surfaces exhibit photocatalytic antibacterial activity under 470 nm visible-light irradiation, suggesting that these materials can function as visible-light-responsive nano-bio interfaces rather than simply serving as surface-modified metallic substrates.^22^ However, previous investigations primarily focused on antimicrobial activity, and it remains unclear whether the same Au-coated NiTi–O nanotube platform can also regulate cellular behavior and enhance osteogenesis under different irradiation wavelengths. Demonstrating wavelength-dependent biological functions using a single Au-coated NiTi–O nanotube platform would establish an important foundation for the development of multifunctional dental metal surfaces.^23^

Photothermal therapy utilizes light-responsive materials that absorb optical energy and generate localized thermal elevation.^24,25^ Although high-intensity photothermal effects can be applied for bacterial eradication or tumor ablation, controlled mild photothermal stimulation at biomaterial surfaces may regulate heat shock protein expression, cytoskeletal tension, mechanobiological signaling, and osteogenic responses without inducing cellular damage.^26–28^ In implant-related applications, photothermal responses are increasingly recognized as a multifunctional surface-engineering strategy capable of simultaneously promoting antimicrobial activity and osteogenic function. Recent studies further suggest that photothermal dental and orthopedic implant surfaces may enable concurrent infection control and enhanced osseointegration.^29–31^

Nevertheless, because 600 nm visible light exhibits limited tissue penetration compared to near-infrared light, caution is required when interpreting whether implant fixtures fully embedded within bone can be directly activated solely through external irradiation.^32–34^ Therefore, the clinical relevance of the present study lies in demonstrating that localized photothermal activity from Au-coated NiTi–O nanotube surfaces can modulate early osteogenic responses in clinically accessible regions, such as exposed implant surfaces, healing abutments, implant collars, or transmucosal components during surgical procedures.

In the present study, we designed an Au-coated NiTi–O nanotube surface as a visible-light-responsive nano-bio interface for regulating the osteogenic activity of human mesenchymal stem cells (hMSCs). Unlike conventional implant surface modifications that primarily rely on passive nanotopographical cues, the present platform combines anodically fabricated NiTi–O nanotubes with an Au nanolayer. This design enables both nanoscale topographical guidance and light-responsive functionality. We hypothesized that 600 nm irradiation would enhance osteogenic differentiation on Au-coated nanotubular surfaces by providing mild photo-mediated stimulation without compromising cytocompatibility. To test this hypothesis, we systematically evaluated surface morphology, hMSC viability, alkaline phosphatase (ALP) activity, osteogenic gene expression, matrix mineralization, and heat shock protein (HSP)-associated cellular responses.

## 2. Materials and methods

### 2.1 Specimen fabrication and surface characterization

All reagents used for the fabrication of NiTi–O nanotubes (NTO) were of ACS grade. Experimental specimens were prepared according to the protocol established in our previous study.^22^ Briefly, NiTi alloy plates (5 × 5 cm^2^, 0.127 mm thick; Alfa Aesar, Ward Hill, MA, USA) were sequentially polished using 600-, 1200-, and 2000-grit silicon carbide papers to reduce surface roughness and remove surface contaminants. The polished plates were anodized in ethylene glycol containing 0.2 w/v% NH_4_F and 1.0 vol% deionized water at 25 V for 1 h. Following anodization, the specimens were rinsed with acetone, ultrasonically cleaned in deionized water, dried overnight at 60 °C, and heat-treated at 400 °C for 3 h to crystallize the nanotubular structure. In terms of the components of anodizing solution, NH_4_F serves as the source of fluoride ions (F^−^), which are essential for the self-organized formation of the nanotubular oxide. During anodization, field-assisted oxide growth at the metal/oxide interface competes with the localized chemical dissolution of the oxide by F^−^, which forms soluble fluoride complexes (*e.g.*, [TiF_6_]^2−^).^35,36^ This dynamic equilibrium between oxide formation and fluoride-induced dissolution, together with the small ionic radius of F^−^ that permits migration through the growing oxide, drives the development of the ordered nanotube array. Because NiTi alloy is chemically less durable than pure Ti, a near-neutral NH_4_F-based electrolyte was used to moderate the fluoride-assisted dissolution rate and thereby achieve fine control over nanotube formation.^9,37^

Au-coated NiTi–O nanotubes were fabricated by ion plasma sputtering (E-1030, Hitachi Co., Tokyo, Japan) for 30 s. The surface morphology of the Au-coated NiTi–O nanotubes was characterized by field-emission scanning electron microscopy (FE-SEM; S-4800, Hitachi Co., Tokyo, Japan), and the elemental distribution was further analyzed by spherical aberration corrected scanning transmission electron microscope (STEM-Cs; JEM-ARM200F, JEOL Co., Tokyo, Japan). Surface chemical states were analyzed by XPS (K-alpha, Thermo Scientific Co., Waltham, USA) with a monochromated Al Kα source (400 µm spot, 50.0 eV pass energy, 0.10 eV step). Binding energies were calibrated to the adventitious C 1s peak at 284.8 eV. Three experimental groups were prepared: untreated NiTi (NiTi), anodized NiTi–O nanotubes (NTO), and Au-coated NiTi–O nanotubes (ANT). For cell-based experiments, each group was further divided into non-irradiated controls and 600 nm LED-irradiated groups. The structural characteristics of the NiTi, NTO, and ANT surfaces were consistent with those reported previously.^22^ Optical absorption properties were also evaluated using diffuse reflectance ultraviolet-visible-near-infrared spectrophotometry, as described in the SI. LED irradiation was delivered using a lab-fabricated device at an intensity of 5.5 mW cm^−2^ with a working distance of 4 cm from the specimen surface.

### 2.2 Live/dead assay

A live/dead assay was performed to evaluate cell viability. Human mesenchymal stem cells (hMSCs; PT-2501, Lonza Ltd., Basel, Switzerland) at passage 4–5 were cultured in α-MEM supplemented with 10% fetal bovine serum (FBS) and 1% penicillin–streptomycin at 37 °C under 5% CO_2_. Since commercially obtained hMSCs were used, this study was exempted from review by the Institutional Review Board of Wonkwang University (WKUIRB 202301-001-01). Cells were seeded onto specimens (1 × 1 cm^2^) placed in 24-well plates at a density of 5 × 10^3^ cells per well and cultured for 24 or 48 h. Experimental groups were irradiated with 600 nm LED light at 5.5 mW cm^−2^ for 10 min daily, beginning 24 h after cell seeding. For fluorescence staining, cells were incubated in PBS containing 2 µM calcein AM and 4 µM ethidium homodimer-1 (EthD-1) for 30 min at 37 °C. Live and dead cells were visualized as green and red fluorescence, respectively, using a fluorescence inverted microscope after 24 and 48 h of culture.

### 2.3 MTT assay

An MTT assay was performed to quantitatively evaluate cytotoxicity using a commercial MTT assay kit under direct-contact conditions according to ISO 10993-5, Annex C.^38^ The same cell culture and LED irradiation conditions used in the live/dead assay were applied. After 24 and 48 h of culture, 1 mL of MTT solution was added to each well and incubated for 4 h at 37 °C under 5% CO_2_. The resulting formazan crystals were dissolved in dimethyl sulfoxide (DMSO), and absorbance was measured at 570 nm using a microplate reader (SpectraMax Mini, Molecular Devices, San Jose, CA, USA). Specimens exhibiting ≥70% cell viability relative to the control group were considered biocompatible according to ISO 10993-5. Six specimens were analyzed per group (*n* = 6).

### 2.4 Alkaline phosphatase (ALP) activity

Osteogenic activity was assessed by measuring ALP, an early marker of osteogenic differentiation. hMSCs were seeded at a density of 2.0 × 10^4^ cells per well and cultured in osteogenic differentiation medium supplemented with 10 mM β-glycerophosphate, 50 µg mL^−1^ ascorbic acid, and 50 nM dexamethasone. During the initial 48 h, the irradiation protocol was identical to that used in the live/dead assay. Thereafter, cells were irradiated for 10 min every 3 days. After 1 and 2 weeks of culture, cells were lysed in assay buffer for 30 min. Cell lysates were incubated with 5 mM *p*-nitrophenyl phosphate (*p*-NPP) solution at 37 °C for 30 min, followed by the addition of stop solution. Absorbance was measured at 405 nm using a microplate reader. ALP activity was normalized to total protein concentration determined using a bicinchoninic acid (BCA) protein assay kit. Owing to the limited specimen size (1 × 1 cm^2^), the amount of cell lysate obtained from a single specimen was insufficient for reliable measurement of ALP activity. Therefore, cells harvested from two specimens were pooled to constitute one biological sample, and ALP activity was measured in four pooled samples per group (*n* = 4).

### 2.5 Quantitative real-time polymerase chain reaction (PCR)

Quantitative real-time PCR was performed to evaluate the expression of osteogenesis-related genes. hMSCs were cultured and irradiated under the same conditions used for the ALP activity assay. Total RNA was extracted from cells cultured on the specimens using TRIzol reagent and reverse-transcribed into complementary DNA (cDNA) using a cDNA synthesis kit. Osteopontin (OPN), osteocalcin (OCN), and bone sialoprotein-2 (BSP-2) expression levels were analyzed after 2 weeks. Real-time PCR was performed using TaqMan® gene expression assays (Applied Biosystems, Foster City, CA, USA), and target gene expression levels were normalized to GAPDH expression. Relative expression was calculated by the 2^−ΔΔ*C*_t_^ method using the non-irradiated NiTi group as the calibrator, which is, therefore, assigned a value of 1.000 by definition. Owing to the limited specimen size (1 × 1 cm^2^), the amount of RNA obtained from a single specimen was insufficient for reliable quantitative analysis. Therefore, cells harvested from two specimens were pooled to constitute one biological sample, and qPCR was performed on 3 pooled samples per group (*n* = 3).

### 2.6 Alizarin Red S (ARS) assay

An Alizarin Red S (ARS) assay was performed to quantify calcium deposition. hMSCs were cultured and irradiated under the same conditions used for the ALP activity assay. After 3 weeks of culture, specimens were washed with PBS, fixed with 4% paraformaldehyde for 20 min, and stained with ARS solution for 20 min. After washing, the bound dye was extracted using 10% acetic acid for 30 min. The solution was then heated at 85 °C for 10 min and centrifuged at 11 000 rpm for 15 min. The supernatant was neutralized with an equal volume of 10% ammonium hydroxide, and absorbance was measured at 405 nm using a microplate reader. Six specimens were included in each group (*n* = 6).

### 2.7 HSP assay

The effects of photothermal stimulation on HSP27 and HSP70 gene expression were evaluated by real-time PCR using a SYBR Green assay (PowerSYBR® Green PCR Master Mix; Applied Biosystems, Foster City, CA, USA). Primer sequences are summarized in Table 1 hMSCs were seeded onto specimens placed in 24-well plates at a density of 1 × 10^4^ cells per well and cultured for 24 h. Cells were subsequently irradiated with 600 nm visible light for 10 min once daily for two consecutive days. Total RNA was extracted 2 h after the second irradiation using TRIzol reagent, and cDNA was synthesized using a cDNA synthesis kit. HSP27 and HSP70 expression levels were normalized to GAPDH expression. Because the number of cells obtained from a single specimen was insufficient, cells from two specimens were pooled into one sample, resulting in three pooled samples per group (*n* = 3).

**Table 1:** Primer sequences used for SYBR green-based real-time PCR analysis of HSP-related genes and the reference gene GAPDH

Gene	Primer sequence (5′ → 3′)
GAPDH	Forward: 5′-ATT TGG TCG TAT TGG GCG-3′
	Reverse: 5′-TGG AAG ATG GTG ATG GGA TT-3′
HSP27	Forward: 5′-GTC CAA CGA GAT CAC CAT CC-3′
	Reverse: 5′-CGG CAG TCT CAT CGG ATT-3′
HSP70	Forward: 5′-TTG CAG TGT GCC ATC TTA TC-3′
	Reverse: 5′-GAG AAA GGA GCA GCA TGA TT-3′

### 2.8 Statistical analysis

Data are presented as mean ± SD and were analyzed using GraphPad Prism version 8.4.3. Each endpoint was evaluated with a 3 × 2 factorial design comprising surface type (NiTi, NTO, ANT) and irradiation (absent or present; 600 nm) as fixed factors and was analyzed by two-way ANOVA using Type III sums of squares. Regardless of the significance of the interaction term, all six treatment combinations were then compared by Tukey's honestly significant difference test based on the pooled residual mean square and residual degrees of freedom of the corresponding two-way model. In the figures, groups not sharing a common letter differ at *P* < 0.05. Full ANOVA output is given in Table 2.

**Table 2:** Two-way ANOVA of surface type and 600 nm visible-light irradiation (type III sums of squares) for the endpoints reported in the main text. The corresponding analysis of the YAP nuclear/cytoplasmic ratio is given in Table S6^a^

Source of variation	df	MS	*F* ratio	*P* value
Cell viability (MTT), 24 h				
Interaction	2	170.6	*F* (2, 30) = 1.491	0.2414
Surface	2	92.79	*F* (2, 30) = 0.8108	0.4540
Irradiation	1	183.2	*F* (1, 30) = 1.601	0.2155
Residual	30	114.5		

Cell viability (MTT), 48 h				
Interaction	2	145.4	*F* (2, 30) = 1.912	0.1654
Surface	2	778.2	*F* (2, 30) = 10.23	0.0004
Irradiation	1	159.6	*F* (1, 30) = 2.098	0.1578
Residual	30	76.07		

ALP activity, 1 week				
Interaction	2	0.5196	*F* (2, 18) = 0.6965	0.5113
Surface	2	0.6773	*F* (2, 18) = 0.9080	0.4210
Irradiation	1	33.59	*F* (1, 18) = 45.03	<0.0001
Residual	18	0.7459		

ALP activity, 2 weeks				
Interaction	2	3.196	*F* (2, 18) = 4.849	0.0207
Surface	2	23.77	*F* (2, 18) = 36.06	<0.0001
Irradiation	1	5.753	*F* (1, 18) = 8.727	0.0085
Residual	18	0.6592		

OPN mRNA, 2 weeks				
Interaction	2	0.2643	*F* (2, 12) = 2.278	0.1450
Surface	2	0.9665	*F* (2, 12) = 8.331	0.0054
Irradiation	1	0.6436	*F* (1, 12) = 5.548	0.0364
Residual	12	0.1160		

OCN mRNA, 2 weeks				
Interaction	2	0.2132	*F* (2, 12) = 4.555	0.0337
Surface	2	1.012	*F* (2, 12) = 21.62	0.0001
Irradiation	1	0.4002	*F* (1, 12) = 8.551	0.0127
Residual	12	0.04680		

BSP-2 mRNA, 2 weeks				
Interaction	2	0.07094	*F* (2, 12) = 3.543	0.0618
Surface	2	0.3055	*F* (2, 12) = 15.26	0.0005
Irradiation	1	0.3412	*F* (1, 12) = 17.04	0.0014
Residual	12	0.02003		

Alizarin red S (ARS), 3 weeks				
Interaction	2	10 694	*F* (2, 30) = 0.6079	0.5511
Surface	2	1 238 955	*F* (2, 30) = 70.43	<0.0001
Irradiation	1	235 387	*F* (1, 30) = 13.38	0.0010
Residual	30	17 591		

HSP70 mRNA, 48 h				
Interaction	2	0.1328	*F* (2, 12) = 4.106	0.0438
Surface	2	0.3021	*F* (2, 12) = 9.344	0.0036
Irradiation	1	0.6115	*F* (1, 12) = 18.91	0.0009
Residual	12	0.03233		

HSP27 mRNA, 48 h				
Interaction	2	0.2507	*F* (2, 12) = 5.993	0.0157
Surface	2	0.5762	*F* (2, 12) = 13.78	0.0008
Irradiation	1	0.6841	*F* (1, 12) = 16.35	0.0016
Residual	12	0.04183		

a Surface: NiTi, NTO, ANT (three levels). Irradiation: without *vs.* with 600 nm visible light (two levels). MS, mean square. *n* = 6 (cell viability, mineralization), *n* = 4 (ALP activity) and *n* = 3 (mRNA expression) per group.

## 3. Results

### 3.1 Surface characterization of anodized and Au-coated NiTi specimens

Fig. 1(A) presents the surface morphology (FE-SEM) of the Au-coated NiTi–O nanotubes. The NTO specimens exhibited a well-defined nanotubular oxide layer on the anodized NiTi surface, confirming successful nanotube formation. The nanotubes had an average outer diameter of 84.95 ± 5.25 nm, wall thickness of 12.45 ± 1.57 nm, and length of 713.5 ± 16.0 nm. Clearly visible nanotube openings further verified successful anodic oxidation. Following Au sputter coating, the ANT specimens retained the overall nanotubular morphology, with an outer diameter of 86.60 ± 5.70 nm and wall thickness of 13.10 ± 2.00 nm. Preservation of the open nanotube mouths after Au deposition indicates that the sputtered Au layer functionalized the nanotubular interface without obscuring the nanoscale topographical features. Although the ANT surface appeared slightly denser than the NTO surface, the characteristic nanotopography remained preserved.

**Fig. 1:**
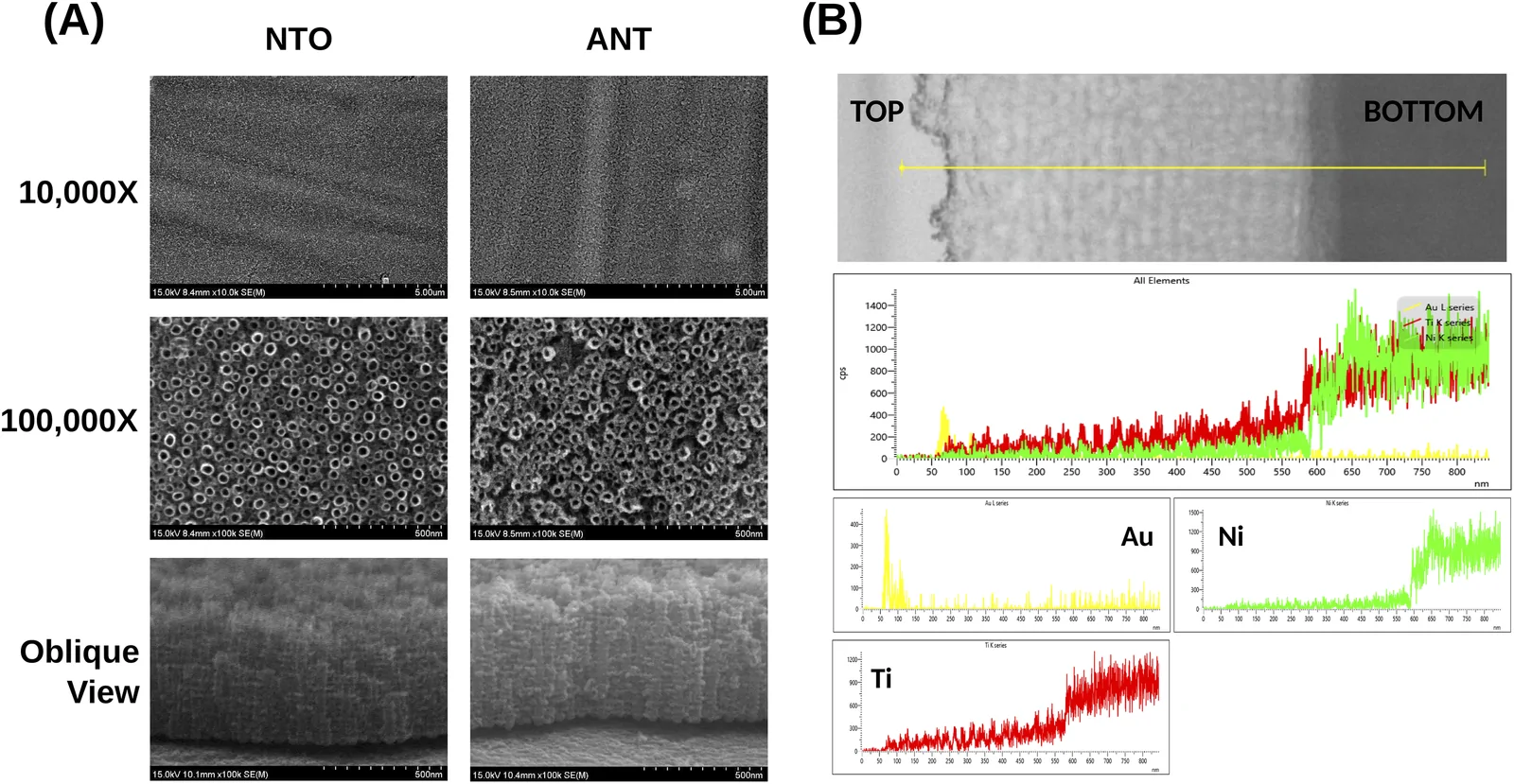
(A) FE-SEM images of anodized NiTi–O nanotubes (NTO) and gold (Au)-coated NiTi–O nanotubes (ANT) (low magnification (10 000×), high magnification (100 000×), and oblique views showing the nanotube structure) and (B) cross-sectional HAADF-STEM image and STEM-EDS line profiles of Au, Ni, and Ti along the depth of a representative region of the Au-coated NiTi–O nanotube layer. The depth scale of the line profile reflects the local thickness of the region analyzed and is not directly comparable with the mean nanotube length determined by FE-SEM.

Fig. 1(B) shows the cross-sectional high-angle annular dark-field (HAADF)-STEM image and STEM-EDS line-scan profiles of the Au-coated NiTi–O nanotube layer. The Au signal exhibited a sharp peak within approximately 100 nm of the surface, the Ti signal increased gradually with depth, and the Ni signal rose abruptly at approximately 600 nm, where a distinct contrast change appeared in the HAADF image, corresponding to the oxide/substrate interface of this cross-section.

XPS confirmed the surface chemical states of the Au-coated NiTi–O nanotubes (Fig. 2). The Au 4f showed a metallic Au^0^ doublet at 84.1 and 87.8 eV, and Ti 2p a Ti^4+^ doublet at 459.0 and 464.8 eV without Ti^0^ or Ti^3+^. O 1s was dominated by lattice oxygen with a hydroxyl (–OH) shoulder, whereas Ni 2p showed only weak intensity. Thus, Au-coated NiTi–O nanotubes comprise a nickel-depleted TiO_2_ nanotubular oxide covered by a metallic Au overlayer, consistent with the STEM-EDS profile (Fig. 1(B)). Notably, the substrate-derived Ti 2p and O 1s signals were still clearly detected together with the Au 4f signal. Because the information depth of Al Kα XPS is limited to approximately 10 nm (≈3*λ*), the metallic Au overlayer covering the nanotube walls must be thinner than this value, that is, of the order of a few nanometers, or locally discontinuous. This upper-bound estimate is consistent with the short sputtering time (30 s), with the preservation of the open nanotube mouths observed by FE-SEM, and with the STEM-EDS profile, in which Au was confined to the uppermost approximately 100 nm of the nanotube array rather than forming a thick continuous film.

**Fig. 2:**
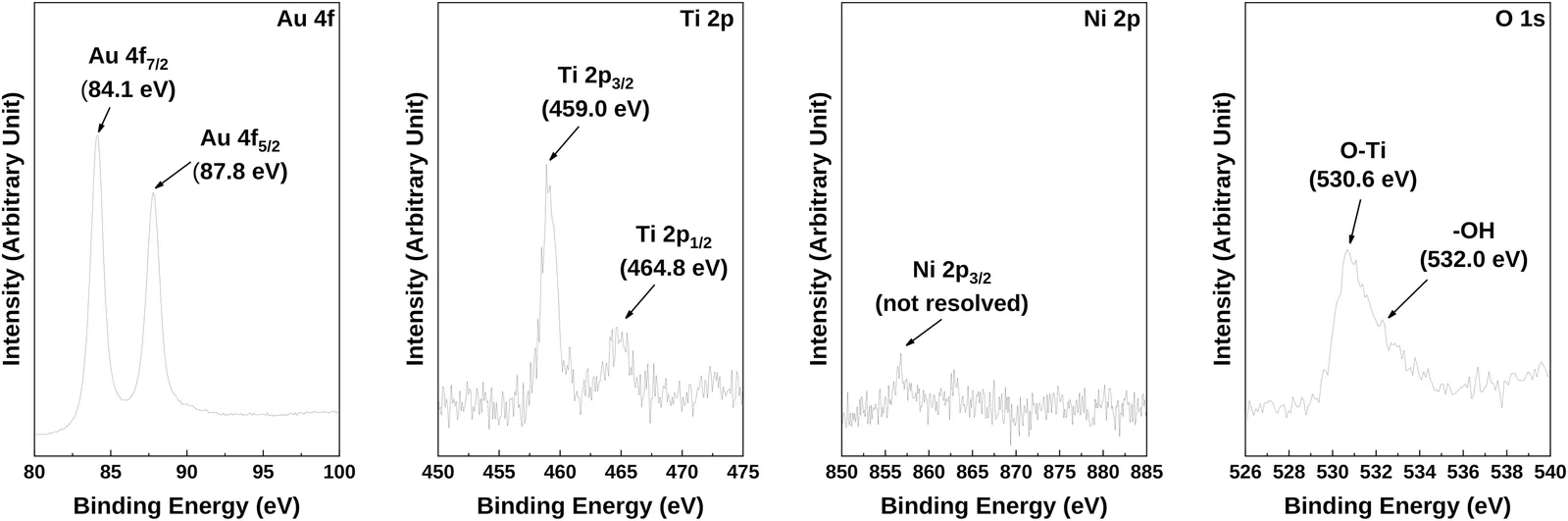
XPS analysis of the Au-coated NiTi–O nanotube (ANT) surface. Au 4f, Ti 2p, Ni 2p, and O 1s core-level spectra. Binding energies were calibrated to the adventitious C 1s peak at 284.8 eV.

These observations confirm that both anodization and subsequent Au deposition were successfully achieved without substantial structural disruption of the nanotube layer. Supplementary diffuse reflectance UV-vis-NIR spectroscopy further demonstrated enhanced optical absorption of the ANT surface. As shown in Fig. S1, ANT exhibited stronger absorption throughout the visible-to-near-infrared region than both NiTi and NTO, particularly near 600 and 780 nm. These findings support that Au coating improved the light-harvesting capability of the nanotubular surface under the 600 nm irradiation conditions used in this study.

### 3.2 Cell viability evaluated by live/dead staining

Live/dead staining demonstrated favorable cytocompatibility of all specimen groups after 24 and 48 h of culture (Fig. 3). At both time points, the majority of hMSCs cultured on all surfaces exhibited green fluorescence, whereas only a few red fluorescent cells were observed. These findings indicate that neither anodization nor Au coating induced significant acute cytotoxicity. In addition, viable cell density appeared greater on nanotubular surfaces. However, the quantitative MTT analysis showed no significant main effect of irradiation at either time point, so this qualitative impression should not be interpreted as an irradiation-dependent proliferative effect.

**Fig. 3:**
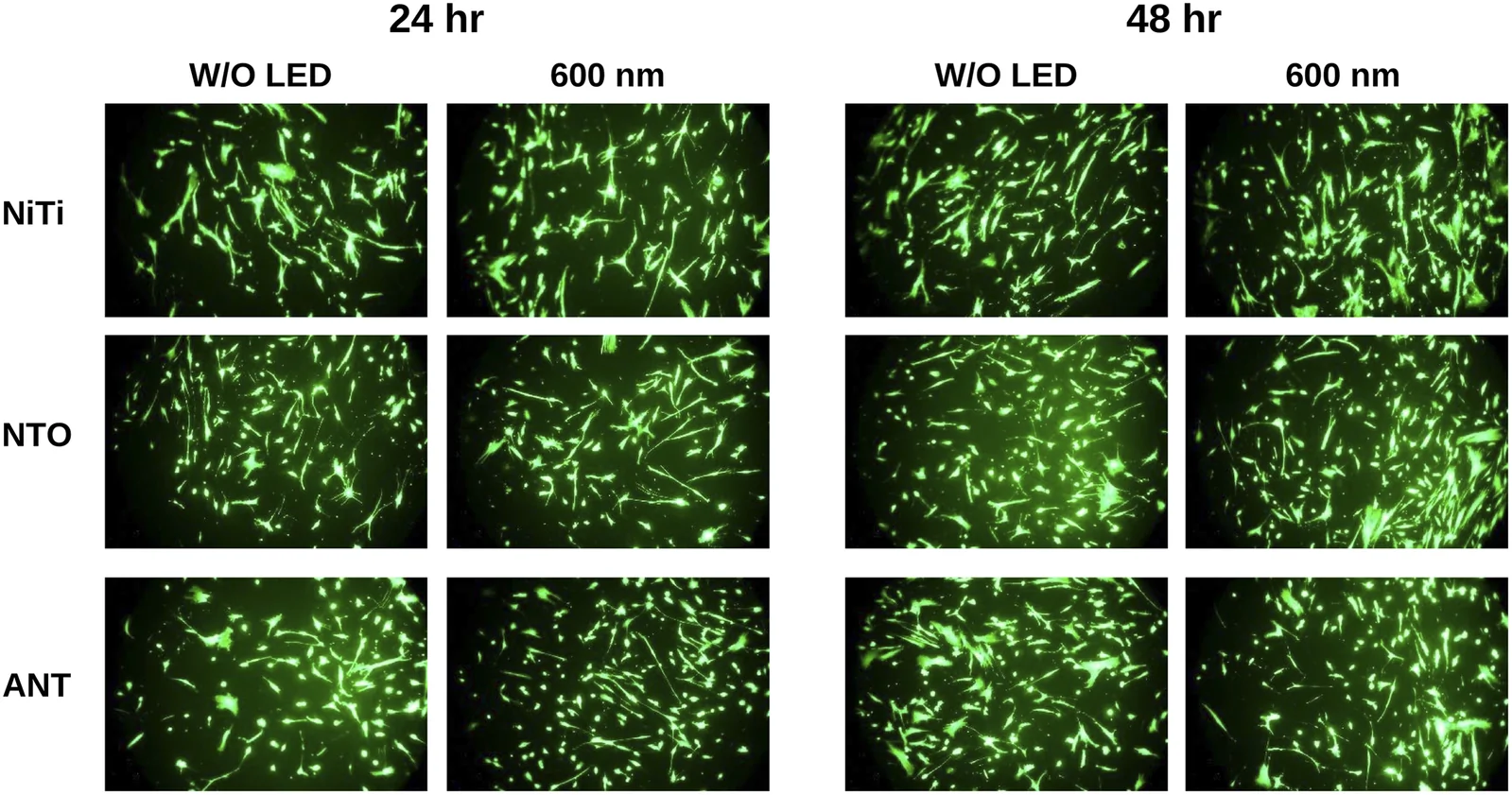
Live/dead fluorescence staining of human mesenchymal stem cells (hMSCs) cultured on NiTi, NTO, and ANT surfaces with or without 600 nm LED irradiation for for 24 and 48 h.

### 3.3 Quantitative assessment of cell viability by MTT assay

The MTT assay further confirmed the biocompatibility of all experimental groups (Fig. 4). After 24 h of culture, cell viability remained comparable to or greater than that of the control group across all specimens, and two-way ANOVA showed no significant main effects of surface type, of 600 nm irradiation, or of their interaction at this time point. These findings indicate that untreated NiTi, NTO, ANT, and their corresponding LED-irradiated groups maintained adequate short-term cellular metabolic activity. Importantly, all viability values remained above the cytocompatibility threshold defined in ISO 10993-5 (dotted line in the graph). After 48 h of culture, two-way ANOVA demonstrated a significant main effect of surface type (*P* = 0.0004), whereas neither irradiation (*P* = 0.1578) nor the surface × irradiation interaction (*P* = 0.1654) was significant (Table 2). Although numerical differences were observed among the six groups, all groups remained above the ISO 10993-5 cytocompatibility threshold.

**Fig. 4:**
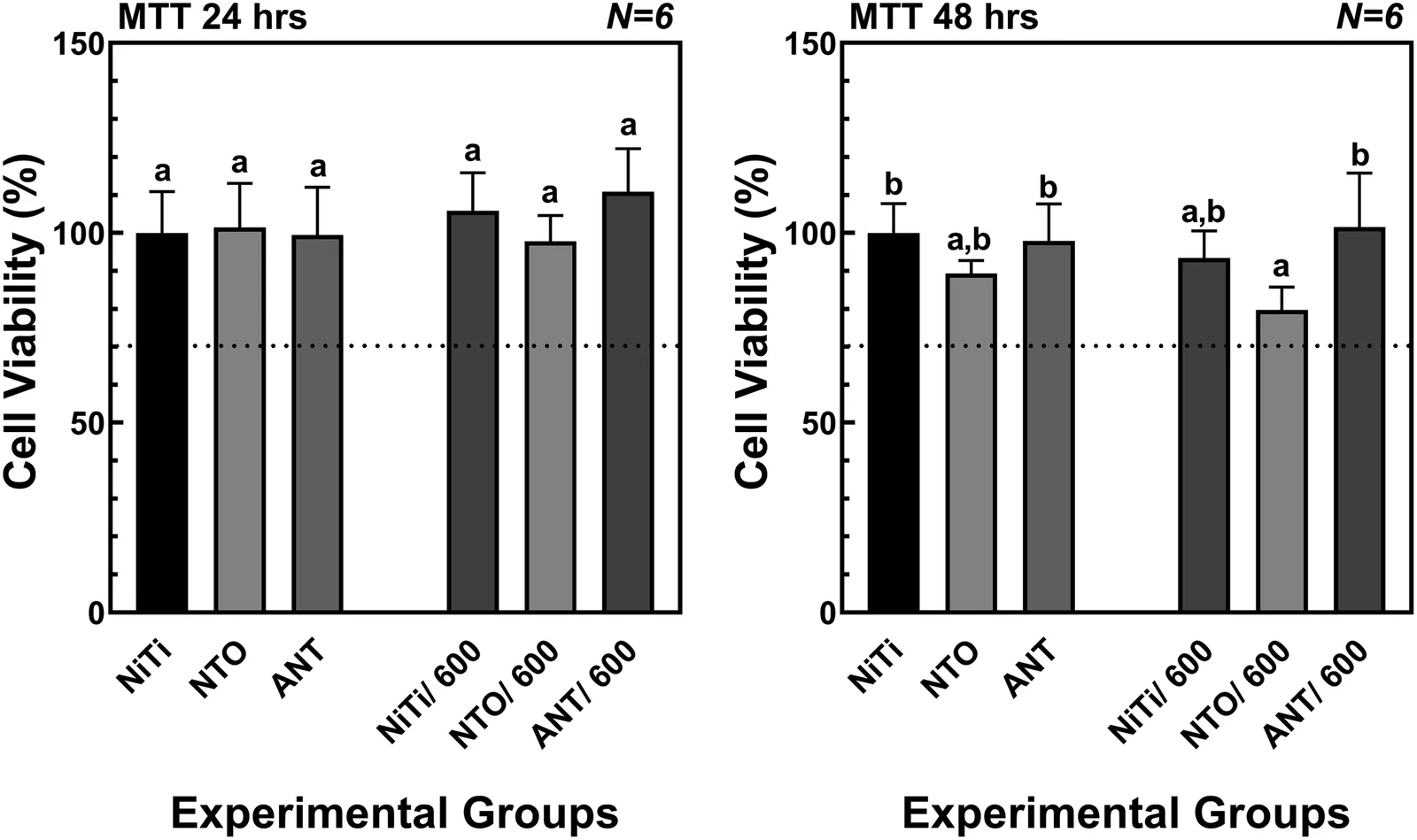
MTT assay results evaluating the metabolic activity and proliferation of hMSCs cultured on NiTi, NTO, and ANT surfaces with or without 600 nm LED irradiation. Data are presented as mean ± standard deviation (*n* = 6). Data were analyzed by two-way ANOVA (surface × irradiation; see Table 2), and groups not sharing a common letter differ at *P* < 0.05 by Tukey's test.

### 3.4 ALP activity

ALP activity was evaluated to assess the early osteogenic response of hMSCs cultured on the different surfaces (Fig. 5). After 1 week, two-way ANOVA showed a significant main effect of irradiation (*P* < 0.0001), whereas the main effect of surface type and the surface × irradiation interaction were not significant. After 2 weeks, both main effects were significant and, importantly, so was the surface × irradiation interaction (*P* = 0.0207) (Table 2). In the six-group Tukey comparison, irradiation significantly increased ALP activity on the NTO surface (*P* = 0.0301), whereas the irradiated and non-irradiated groups did not differ on NiTi or ANT. By this time point the response to visible light had therefore become conditional on the presence of a nanotubular oxide layer, rather than acting uniformly across substrates as it did at 1 week.

**Fig. 5:**
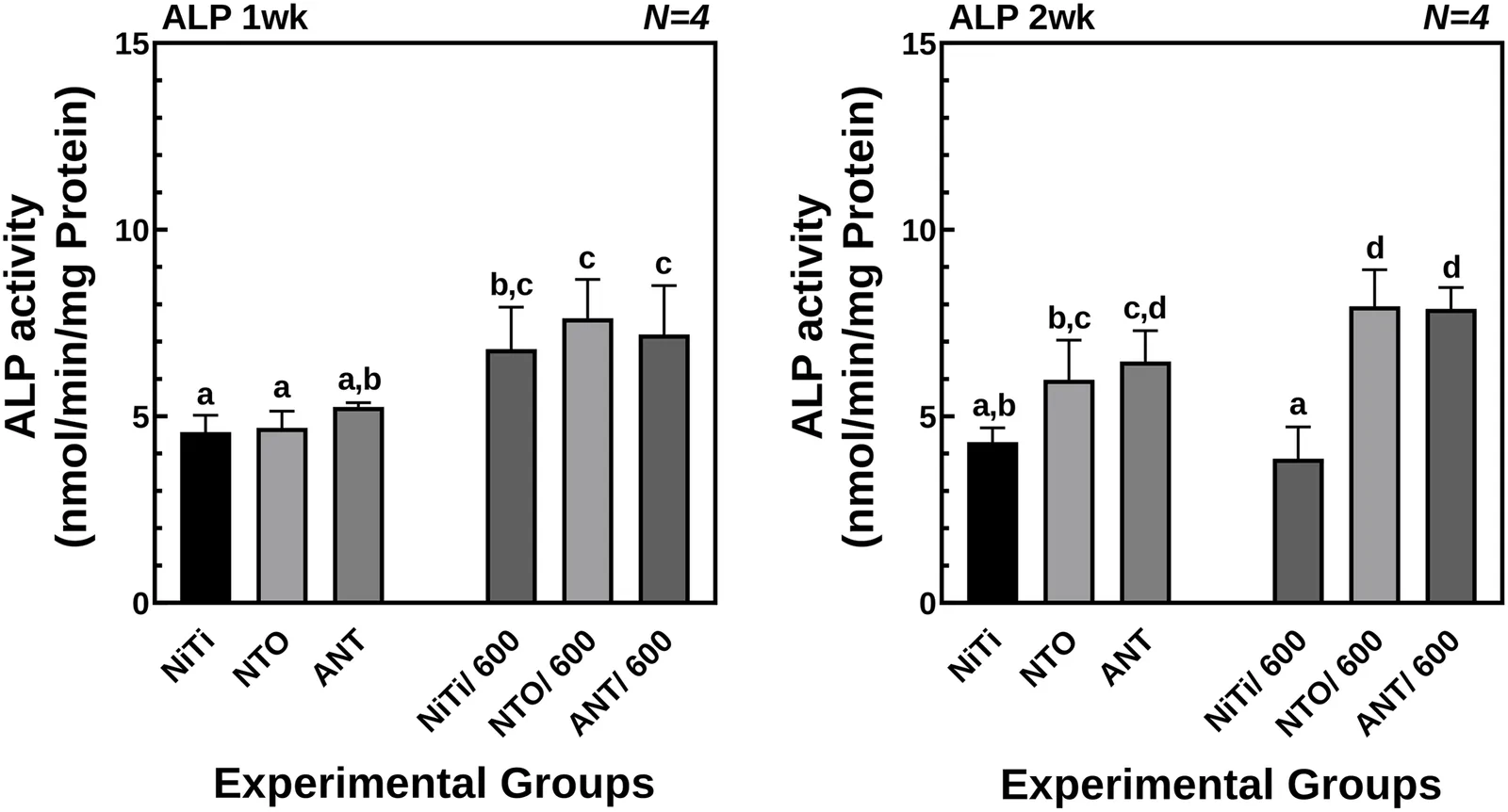
Alkaline Phosphatase (ALP) activity of hMSCs cultured on NiTi, NTO, and ANT surfaces with or without 600 nm LED irradiation. Data are presented as mean ± standard deviation (*n* = 4). Data were analyzed by two-way ANOVA (surface × irradiation; see Table 2), and groups not sharing a common letter differ at *P* < 0.05 by Tukey's test.

### 3.5 Osteogenic differentiation-related gene expression

The expression of osteogenesis-related genes was evaluated by quantitative real-time PCR after 2 weeks (Fig. 6). Two-way ANOVA showed significant main effects of surface type and irradiation for OPN, OCN, and BSP-2. A significant surface × irradiation interaction was observed for OCN (*P* = 0.0337), but not for OPN (*P* = 0.1450) or BSP-2 (*P* = 0.0618) (Table 2). For OPN, ANT/600 showed the highest mean expression and was significantly higher than NiTi and NiTi/600, but was not significantly different from NTO, ANT, or NTO/600. For OCN, ANT/600 and NTO/600 showed the highest expression and did not differ from each other, and both were significantly higher than NiTi and NiTi/600. Within each surface, however, the irradiated and non-irradiated groups did not differ significantly. Therefore, the interaction was driven mainly by the divergent behavior of NiTi relative to the two nanotubular surfaces. For BSP-2, ANT/600 was significantly higher than all other groups, while the corresponding irradiated *versus* non-irradiated differences were not significant for NiTi or NTO.

**Fig. 6:**
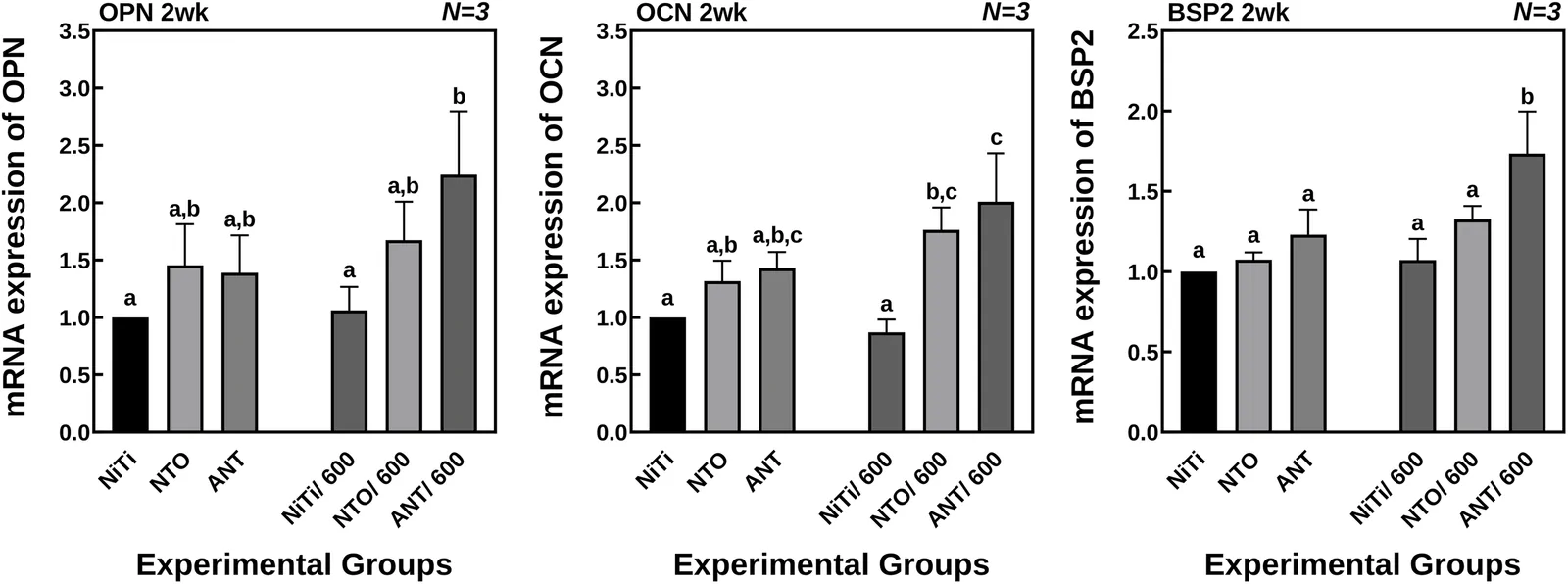
Quantitative real-time RT-PCR analysis of osteogenic differentiation marker genes in hMSCs cultured on NiTi, NTO, and ANT surfaces with or without 600 nm LED irradiation. Data are presented as mean ± standard deviation (*n* = 3). Data were analyzed by two-way ANOVA (surface × irradiation; see Table 2), and groups not sharing a common letter differ at *P* < 0.05 by Tukey's test.

### 3.6 Matrix mineralization evaluated by Alizarin Red S (ARS) assay

Matrix mineralization was quantified after 3 weeks of osteogenic induction using Alizarin Red S (ARS) staining (Fig. 7). Among the experimental groups, the untreated NiTi showed the lowest degree of mineral deposition. The NTO group exhibited greater mineralization than the NiTi group, indicating that the nanotubular surface alone promoted osteogenic maturation. Compared with NTO, the ANT group exhibited a further increase in mineralization, suggesting that Au coating further enhanced matrix mineralization. Two-way ANOVA revealed significant main effects of surface type (*P* < 0.0001) and irradiation (*P* = 0.0010), whereas the surface × irradiation interaction was not significant (*P* = 0.5511) (Table 2). This indicates that irradiation exerted an overall additive effect on mineralization across the three surface conditions. In the six-group Tukey comparison, ANT/600 showed the highest numerical mineralization and was significantly higher than NiTi, NTO, NiTi/600, and NTO/600, but not significantly different from ANT.

**Fig. 7:**
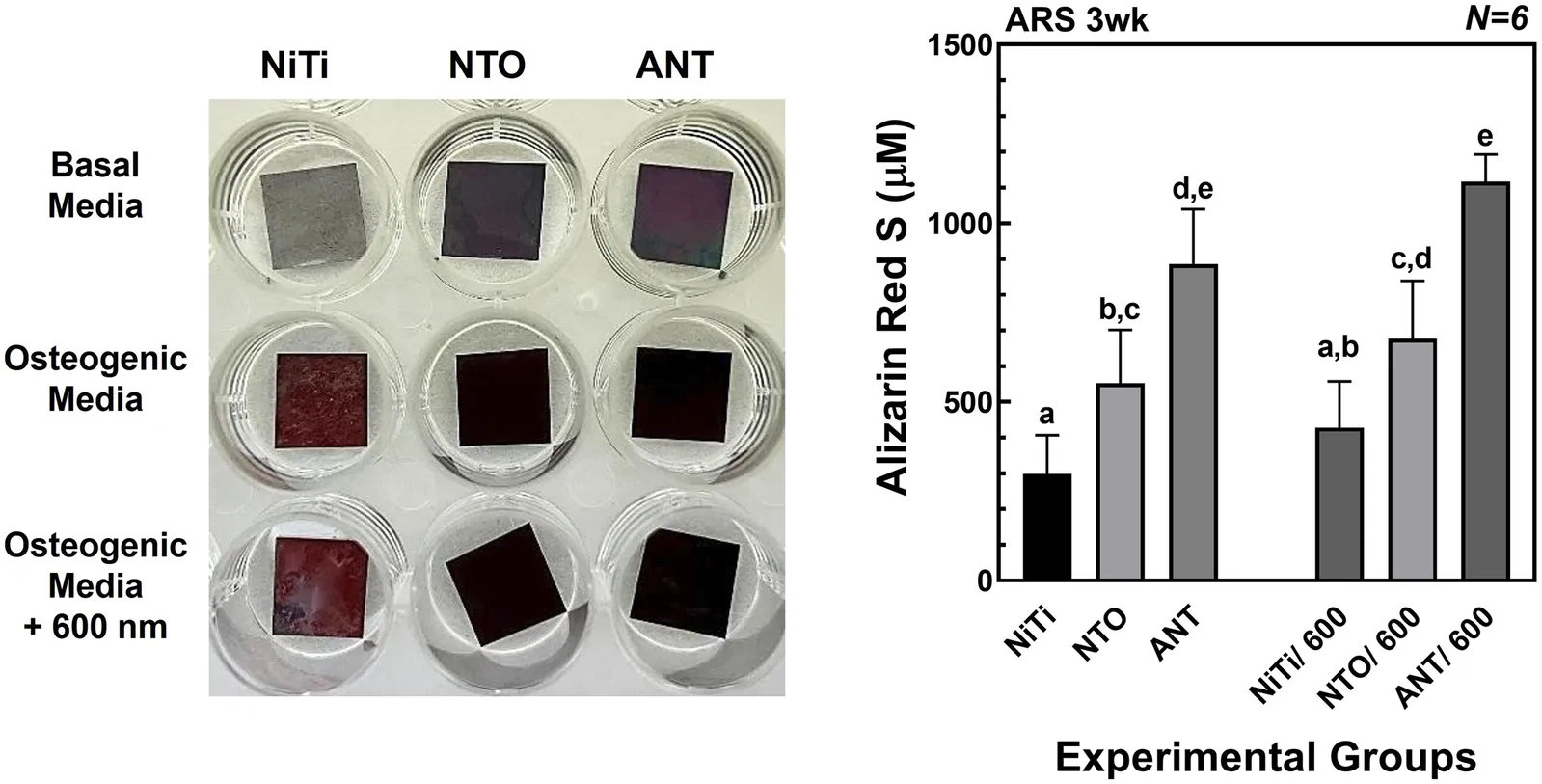
Alizarin Red S (ARS) staining images (left) and quantitative analysis (right) to evaluate calcium deposition and matrix mineralization of hMSCs cultured on NiTi, NTO, and ANT surfaces with or without 600 nm LED irradiation. Data are presented as mean ± standard deviation (*n* = 6). Data were analyzed by two-way ANOVA (surface × irradiation; see Table 2), and groups not sharing a common letter differ at *P* < 0.05 by Tukey's test.

### 3.7 HSP-related gene analysis

To investigate photothermal stimulation-related signaling under the combined effects of nanotopography and visible-light irradiation, the expression levels of HSP27 and HSP70 were analyzed (Fig. 8). For both HSP27 and HSP70, two-way ANOVA revealed significant main effects of surface and irradiation, as well as significant surface × irradiation interactions (Table 2). In the six-group Tukey comparisons, ANT/600 was the only group with significantly higher expression than all five other groups. Pairwise comparison within each surface condition showed that 600 nm irradiation significantly increased HSP27 and HSP70 expression on ANT, but not on NiTi or NTO. The significant interactions indicate that the irradiation-associated HSP response depended on surface type and was observed specifically on the Au-coated nanotubular surface.

**Fig. 8:**
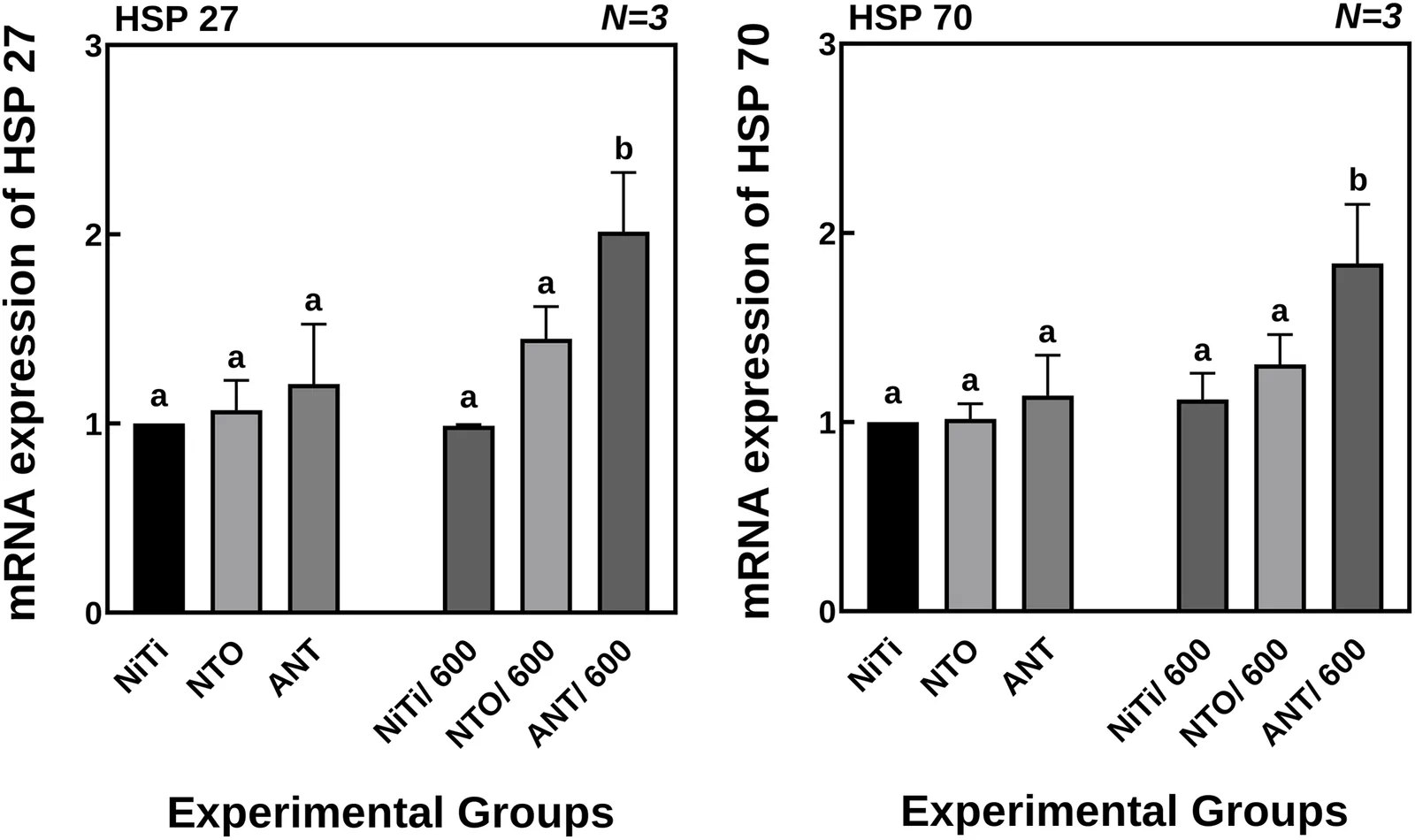
Quantitative real-time RT-PCR analysis of Heat Shock Protein (HSP)-related genes in hMSCs cultured on NiTi, NTO, and ANT surfaces with or without 600 nm LED irradiation. Data are presented as mean ± standard deviation (*n* = 3). Data were analyzed by two-way ANOVA (surface × irradiation; see Table 2), and groups not sharing a common letter differ at *P* < 0.05 by Tukey's test.

## 4. Discussion

This study indicates that Au-coated NiTi–O nanotubes may serve as a visible-light-responsive osteogenic nano-bio interface rather than solely serving as a passive metallic implant surface. The principal finding of this study is that the combination of preserved nanotubular architecture, Au surface functionalization, and 600 nm irradiation enhanced ALP activity, osteogenic gene expression, matrix mineralization, and HSP-related cellular responses without compromising hMSC viability. These results suggest that osteogenic activity at the metal–cell interface can be actively regulated through the integration of nanoscale topographical cues and external optical stimulation.

FE-SEM analysis confirmed that the NTO group exhibited a uniform nanotubular structure and the nanotube apertures and overall structure on the ANT surface were preserved even after Au sputter coating. These findings indicate that Au deposition functionalized the surface without obstructing or collapsing the nanotubular structure. The bioactivity of NiTi implants is known to be strongly influenced by surface oxide chemistry, roughness, and nanoscale morphology.^39,40^ Nanotubular surfaces have been reported to enhance protein adsorption, cell adhesion, cytoskeletal organization, and osteogenic signaling in hMSCs,^41,42^ which may explain the enhanced osteogenic responses observed in the NTO and ANT groups compared with untreated NiTi. XPS further showed that the ANT surface consists of a nickel-depleted TiO_2_ oxide covered by metallic Au^0^, indicating that anodic oxidation and Au coating together limit nickel exposure at the cell-contacting interface.

The live/dead staining and MTT assay results demonstrated that all experimental groups satisfied the cytocompatibility criteria defined by ISO 10993-5 and that 600 nm LED irradiation did not induce acute cytotoxicity. Notably, the ANT/600 group exhibited relatively high metabolic activity after 48 h, indicating that the combination of Au-coated nanotubes and visible-light irradiation did not adversely affect hMSC survival or proliferation. These findings suggest that the 600 nm irradiation parameters used in this study (5.5 mW cm^−2^) were consistent with mild photothermal stimulation within a biocompatible range rather than cytotoxic hyperthermic stress. In contrast to high-intensity photothermal therapy, which induces cellular damage, mild photothermal stimulation has been reported to modulate cytoprotective responses, mechanobiological signaling, and osteogenic activity.^26,43^

Additional surface-temperature measurements further support the interpretation that the 600 nm irradiation conditions used in this study induced a photothermal response. As shown in Fig. S2, the surface temperatures of the NTO and ANT specimens gradually increased with irradiation time, and infrared thermal imaging revealed localized heat accumulation around the specimen surfaces. Notably, the ANT group reached 45.9 °C after 15 min of irradiation, indicating the potential to reach temperatures associated with thermal tissue damage. Therefore, a 10 min irradiation duration was selected to provide photothermal stimulation while minimizing the risk of cellular damage. These results support the possibility that the increased HSP27 and HSP70 expression observed in the ANT/600 group was associated, at least in part, with a mild photothermal response induced by the Au-coated nanotubular surface.

The structural and chemical stability of the Au-coated nanotube interface was also examined under the same irradiation regimen used in the cell experiments. ICP-MS analysis of the immersion medium showed that Ni release from ANT was lower than that from NTO at both 7 and 14 days, and that Au release was small and did not increase between the two time points (Fig. S3A). FE-SEM confirmed that the nanotube array remained intact, with no delamination, cracking, or coalescence of the Au layer into discrete particles (Fig. S3B). The Au-coated nanotube layer therefore remained structurally and chemically stable over the culture period. Electrochemical corrosion testing was not carried out in the present study. Instead, corrosion-related behavior was evaluated by immersion-based ion release performed in the same medium and at the same temperature and irradiation schedule as the cell experiments, so that the measured values reflect the amount of Ni to which the cells were exposed rather than an accelerated polarization response. A complete electrochemical characterization of the ANT surface therefore remains to be established.

To examine whether the photothermal component alone could account for the enhanced HSP responses, 600 nm irradiation was compared with a temperature-matched thermal stimulus (42 °C, approximating the 42.3 °C recorded after 10 min of irradiation) applied on the same schedule without light. Relative to 37 °C, irradiation increased HSP70 expression, whereas the increase under 42 °C incubation did not reach significance, and a broadly comparable pattern was observed for ALP activity and mineralization (Fig. S4 and S5, Tables S1–S4). The two stimuli did not differ significantly from each other, however, and a response to irradiation was also observed in the specimen-free osteogenic control. These observations are therefore not sufficient to attribute the enhanced responses exclusively to a surface-mediated photothermal effect, and they suggest that a direct photobiological action of 600 nm light on the cells may contribute alongside any surface-mediated component. Because the three culture conditions formed a graded rather than a categorical series, the relative contributions of these two pathways could not be separated quantitatively within the present design, and the above interpretation should therefore be regarded as tentative.

ALP activity, a representative marker of early osteogenic differentiation, showed an increasing trend in the 600 nm irradiation groups at both 1 and 2 weeks. ALP activity was highest in the NTO/600 and ANT/600 groups, indicating that the combination of nanotubular topography and photostimulation may have contributed to enhanced early osteogenesis. TiO_2_-based nanotubular surfaces have been reported to promote osteogenesis by regulating mechanobiological pathways, including YAP and Piezo1 signaling,^8^ and NiTi–O nanotubes have similarly been proposed as effective nanostructured platforms for modulating cellular responses.^9^ ALP activity also increased in the NTO/600 group without Au coating. However, the ANT/600 group exhibited more pronounced effects in the OPN, OCN, and BSP-2 and matrix mineralization analyses. The two-way analysis reveals a temporal transition. At week 1, irradiation raised ALP similarly across all surfaces with no interaction, consistent with a substrate-independent photoresponse. By 2 weeks, a significant interaction emerged, with irradiation responsiveness retained only on nanotubular surfaces, indicating dependence on sustained oxide-layer cues.

Analysis of osteogenic gene expression showed that ANT/600 showed the highest expression of OPN, OCN, and BSP-2 genes associated with cell–matrix interaction, late-stage osteogenic differentiation, and matrix mineralization, respectively. Their simultaneous upregulation suggests that Au-coated nanotubes combined with 600 nm irradiation promoted late-stage osteogenic maturation in addition to the early ALP response. However, a significant surface-dependent irradiation effect was confirmed only for OCN, as the interaction terms for OPN and BSP-2 were not significant.

The finding that matrix mineralization was the highest in the ANT/600 group after 3 weeks of the Alizarin Red S (ARS) analysis further supports these molecular findings at the functional level. The highest mean mineralization was observed in ANT/600, which was significantly higher than all groups except ANT. Two-way ANOVA showed significant main effects of surface (*P* < 0.0001) and irradiation (*P* = 0.0010), but no interaction (*P* = 0.5511), indicating additive effects rather than synergistic effects on mineralization. In contrast, osteogenic transcript responses were more surface-dependent, with the strongest response observed on the Au-coated nanotubular surface.

Another important finding of this study was the increased expression of HSP27 and HSP70. Elevated expression of these genes in the ANT/600 group was associated with the Au-coated nanotube interface under 600 nm irradiation, which may reflect the involvement of adaptive cellular responses.^26,27^ HSP27 is primarily involved in actin cytoskeleton regulation, whereas HSP70 contributes to cytoprotection and cell survival signaling. The markedly higher expression of both genes in the ANT/600 group suggests that the Au-coated nanotube surface may have interacted with 600 nm light to produce localized photothermal and photobiological stimulation, which may in turn be involved in adaptive signaling and osteogenic cellular responses.

The supplementary YAP analysis showed that the nanotubular surface structure was closely associated with mechanobiological signaling markers in hMSCs. As shown in Fig. S6 and Tables S5–S7, two-way ANOVA of the nuclear-to-cytoplasmic YAP ratio revealed a significant main effect of surface type (*P* < 0.0001) but no effect of 600 nm irradiation (*P* = 0.1044) and no interaction between the two factors (*P* = 0.9087). The NTO, ANT, NTO/600, and ANT/600 groups exhibited significantly higher ratios than the NiTi and NiTi/600 groups (all adjusted *P* ≤ 0.0004) and were accompanied by more pronounced F-actin organization, whereas ANT did not differ from NTO (*P* = 0.9395). These findings are consistent with the possibility that YAP nuclear translocation and cytoskeletal reorganization were primarily influenced by the physical nanotopographical cues of the nanotube surface rather than by light irradiation. These findings suggest that the nanotopography-associated increase in YAP nuclear localization contributed to the osteogenic response of the nanotubular surfaces, but did not account for the additional enhancement observed in ANT/600, although the causal role of YAP-mediated mechanotransduction was not directly examined. Mild photothermal and HSP-related responses associated with Au coating and 600 nm irradiation may have provided additional effects.^8,26,27^

Due to their surface plasmon resonance properties, Au-based nanostructures can absorb light at specific wavelengths and convert it into heat.^24,44,45^ Consistent with this mechanism, supplementary diffuse reflectance spectroscopy (DRS) analysis showed that ANT exhibited stronger absorption in the visible-to-near-infrared region than NiTi and NTO, with prominent absorption peaks near 600 and 780 nm (Fig. S1). This optical absorption profile is consistent with the enhanced photothermal responsiveness of ANT under 600 nm irradiation. Previous studies have also reported that Au-coated NiTi–O nanotube surfaces exhibit photocatalytic antimicrobial activity under 470 nm visible light irradiation,^22^ while Au- and Pt-multicoated TiO_2_ nanotubes have been shown to simultaneously promote visible-light-mediated antibacterial activity and osteogenic functions.^23^ The present study extends photoresponsive metal nanosurfaces to the regulation of hMSC osteogenic differentiation under 600 nm irradiation. However, compared with near-infrared light, 600 nm visible light exhibits limited penetration through soft and mineralized tissues. Therefore, the present strategy should not be interpreted as an approach for transcutaneously activating the entire surface of a fully embedded implant fixture.^32–34^ Instead, this approach is more applicable to light-accessible implant regions, such as transmucosal components, healing abutments, exposed implant collars, or surgically exposed implant surfaces.

This study has several limitations. First, because hMSCs were evaluated under *in vitro* conditions, the results do not fully replicate the complex oral environment, which includes blood, immune cells, bacterial biofilms, and mechanical loading. Second, additional investigations are required to quantitatively correlate localized surface temperature changes induced by 600 nm irradiation with cellular-level heat transfer and biological responses. Third, the number of biological replicates for the gene-expression endpoints (*n* = 3) provides limited statistical power to detect interaction effects. Fourth, HSP- and YAP-related findings, obtained transcriptionally and by immunofluorescence, remain correlative. YAP inhibition/knockdown and western blotting would be helpful to establish causality with osteogenic enhancement. In addition, the YAP quantification was based on single-cell immunofluorescence in 10 cells per group and showed considerable cell-to-cell variability, so these data should be regarded as supportive rather than definitive. Fifth, irradiation heats the specimen surface locally and gradually, whereas 42 °C incubation warms the whole well uniformly, and the cultures with and without a specimen also differ in substrate. Sixth, the corrosion behavior of the ANT surface was evaluated only by immersion-based ion release under culture-relevant conditions. Electrochemical characterization such as potentiodynamic polarization or electrochemical impedance spectroscopy was not performed and would be required to establish the long-term corrosion resistance of the surface. Nevertheless, this study demonstrated that Au-coated NiTi–O nanotube surfaces, when combined with 600 nm visible-light irradiation, promoted osteogenic differentiation and matrix mineralization of hMSCs while maintaining biocompatibility. These findings provide a useful foundation for the development of photo-responsive dental metal implant surfaces.

## 5. Conclusions

In conclusion, this study indicates that Au-coated NiTi–O nanotubes can act as a visible-light-responsive osteogenic nano-bio interface when exposed to 600 nm irradiation. The Au-coated nanotubular surface maintained cytocompatibility while being associated with enhanced ALP activity, osteogenic gene expression, matrix mineralization, and HSP-associated adaptive responses in hMSCs. These findings suggest that integrating nanoscale surface engineering with light-mediated activation represents a promising approach for developing bioactive metallic implant interfaces, particularly for light-accessible dental and orthopedic applications. Nevertheless, further investigations involving protein-level mechanistic analyses and *in vivo* osseointegration models are required to elucidate the underlying photo-mediated mechanisms and evaluate their translational potential.

## Conflicts of interest

The authors declare that they have no known competing financial interests or personal relationships that could have influenced the work reported in this study.

## Data Availability

The data supporting the findings of this study are available from the corresponding author upon reasonable request. Supplementary information (SI): supplementary methods and results, including diffuse reflectance UV-vis-NIR spectra (Fig. S1), surface-temperature measurements under 600 nm irradiation (Fig. S2), ICP-MS ion-release and FE-SEM stability data (Fig. S3), temperature-matched thermal-control experiments for HSP expression, ALP activity and mineralization (Fig. S4, S5 and Tables S1–S4), and YAP immunofluorescence with full statistical analyses (Fig. S6 and Tables S5–S7). See DOI: https://doi.org/10.1039/d6ra04604e.
